# Retraction for Somatic mutation of the cohesin complex subunit confers therapeutic vulnerabilities in cancer

**DOI:** 10.1172/JCI202472

**Published:** 2026-01-16

**Authors:** Yunhua Liu, Hanchen Xu, Kevin Van der Jeught, Yujing Li, Sheng Liu, Lu Zhang, Yuanzhang Fang, Xinna Zhang, Milan Radovich, Bryan P. Schneider, Xiaoming He, Cheng Huang, Chi Zhang, Jun Wan, Guang Ji, Xiongbin Lu

Original citation: *J Clin Invest*. 2018;128(7):2951–2965. https://doi.org/10.1172/JCI98727

Citation for this retraction: *J Clin Invest*. 2026;136(1):e202472. https://doi.org/10.1172/JCI202472

Indiana University, The Ohio State University, and the University of Maryland, College Park jointly notified the *JCI* of data falsification in Supplemental Figure 8A and Supplemental Figure 9E and misconduct findings against Xiongbin Lu and Yunhua Liu. In accordance with the investigation committee’s recommendation, the *JCI* is retracting this article.

An independent investigation by Longhua Hospital, Shanghai University of Traditional Chinese Medicine is ongoing.

Yunhua Liu, Hanchen Xu, Xinna Zhang, Xiaoming He, and Xiongbin Lu dissent from the Journal’s decision to retract. The remaining authors abstained from commenting or could not be reached.

## Version 1 01/16/2026

Electronic publication

